# Wound Healing Activity of *Uncaria callophylla* Stem Methanol Extract in Diabetic Rats and Its Phytochemical Profile

**DOI:** 10.21203/rs.3.rs-8445387/v1

**Published:** 2026-01-05

**Authors:** Astri Rozanah Siregar

**Affiliations:** IPB University

**Keywords:** Uncaria callophylla, diabetic wound healing, topical ointment, phenolic compounds, antioxidant activity, LC-MS/MS, GC-MS

## Abstract

**Background:**

Impaired wound healing is a major complication in diabetic patients. Currently available wound therapies may cause adverse effects, highlighting the need for safer, natural alternatives. This study evaluated the wound healing potential of *Uncaria callophylla* Blume ex Korth. stem methanol extract (UCSME) in diabetic rats.

**Methods:**

Diabetic excision wound models were established and animals were divided into six groups: non-diabetic untreated wounds, diabetic untreated wounds, diabetic wounds treated with UCSME ointment at concentrations of 5%, 10%, and 20%, and diabetic wounds treated with betadine ointment. Wound healing was assessed through wound area reduction, epithelialization time, inflammatory and proliferative cell counts, angiogenesis, collagen deposition, and hydroxyproline content. Phytochemical composition and antioxidant activity were also analyzed.

**Results:**

UCSME ointment significantly accelerated wound closure, shortened epithelialization time, enhanced angiogenesis, re-epithelialization, collagen synthesis, and increased neutrophil, macrophage, and fibroblast counts compared to untreated diabetic controls (p < 0.05). A 5% concentration was sufficient to improve wound closure and proliferative cell responses, while 10% and 20% concentrations showed stronger effects on inflammatory response and angiogenesis. The 20% UCSME ointment produced the most pronounced improvements in angiogenesis, re-epithelialization, and hydroxyproline levels. UCSME contained high total phenolic (20.99%) and flavonoid (0.40%) contents and exhibited strong antioxidant activity (IC_50_ = 16.06 ppm). LC-MS/MS and GC-MS analyses identified several bioactive compounds associated with wound healing and antioxidant effects.

**Conclusion:**

Topical UCSME effectively promotes wound healing in diabetic rats and represents a promising natural therapeutic candidate for diabetic wound management.

## INTRODUCTION

Wound healing is a complex biological process involving four overlapping phases: hemostasis, inflammation, proliferation, and maturation. After injury, platelet aggregation prevents bleeding, followed by neutrophil infiltration to remove debris and prevent infection. Neutrophils differentiate into macrophages, which regulate inflammation and tissue repair. During the proliferative phase, keratinocyte migration, angiogenesis, and fibroblast activation lead to granulation tissue formation, while remodeling restores extracellular matrix integrity and closes the wound (Nasiry *et al*. 2022).

Diabetes mellitus is a rapidly growing global health problem and is frequently associated with impaired wound healing, chronic ulceration, and limb amputation (Okonkwo dan Dipietro 2017). The World Health Organization predicts that the global prevalence of diabetes will reach 693 million by 2045, making diabetic wounds a major clinical and socioeconomic burden. Approximately 25% of diabetic patients experience delayed wound healing, often resulting in infection, amputation, and reduced quality of life (Burgess *et al*. 2021).

Delayed healing in diabetic wounds is driven by chronic hyperglycemia, oxidative stress, impaired angiogenesis, delayed collagen synthesis, and fibroblast dysfunction. Hyperglycemia causes endothelial dysfunction, reduced capillary density, and impaired nutrient delivery to wound tissue. In addition, oxidative stress and biofilm formation disrupt inflammatory and proliferative responses, leading to chronic non-healing wounds (Tan *et al*. 2019; Zandifar *et al*. 2012; Sinaga *et al*., 2019).

Given the limitations and side effects of current therapies, plant-based treatments with antioxidant and regenerative properties are increasingly explored. *Uncaria callophylla* Blume ex Korth., called *bajakah* by local Dayak community in Central Kalimantan, traditionally used in wound treatment, contains bioactive compounds with potential antioxidant and wound-healing activities. Therefore, this study aimed to evaluate the effectiveness of topical *Uncaria callophylla* stem methanol extract (UCSME) ointment on diabetic wound healing in rats by assessing macroscopic healing, histopathological changes, hydroxyproline content, antioxidant activity, and phytochemical composition.

## MATERIAL AND METHODS

### Plant Material and Identification

*Uncaria callophylla* stem samples were collected from tropical peat swamp forests in Central Kalimantan, Indonesia. Botanical identification was conducted at the Bogoriense Herbarium, Research Center for Biology, BRIN (Cibinong, Indonesia), to confirm species authenticity.

### Preparation of *Uncaria callophylla* Stem Methanol Extract (UCSME)

Stems were air-dried at 50 °C, pulverized, and 500 g of powder was macerated in methanol (1:8 w/v) for 72 h with orbital shaking. The filtrate was concentrated using a rotary evaporator to obtain a viscous extract, which was stored at 5 °C until use.

### Experimental Animals and Ethical Approval

Male Sprague Dawley rats (180–200 g) were obtained from Iratco Laboratory (Bogor, Indonesia). All experimental procedures were approved by the Iratco Laboratory Ethics Committee (No. 4.2.004–1/KEHI/I/2023 and 4.2.005–1/KEHI/I/2023).

### Induction of Diabetes

After a 14-day acclimatization period, diabetes was induced by a single intraperitoneal injection of streptozotocin (65 mg/kg BW) as done by other researchers (Furman 2021 and Ozay *et al*. 2013). Rat with fasting blood glucose levels >200 mg/dL after 10 days were considered diabetic and included in the study (Furman, 2021).

### Excision Wound Model

Rats were anesthetized with ketamine-xylazine (1:1; 5 mg/kg BW). A full-thickness excision wound (2 cm diameter) was created on the dorsal surface using a sterile biopsy punch as done by other researchers with slight modifications (Sahoo *et al*. 2015; Nagar *et al*. 2016; Tuhin *et al*. 2017; Nasiry *et al*. 2022).

### Ointment Formulation and Treatment

UCSME ointments (5%, 10%, and 20% w/w) were prepared using a petroleum jelly-lanolin base (1:1) as used by other researchers (Ozay *et al*. 2013 and Yassine *et al*. 2020). Treatments were applied topically three times daily until complete wound closure. Betadine ointment served as the reference treatment.

### Wound Healing Assessment

Wound area reduction was measured every three days using transparent tracing and millimeter paper, and expressed as percentage reduction. Complete epithelialization time was recorded as the number of days required for total wound closure without scab (Nagar *et al*. 2016).

### Histopathological and Biochemical Analyses

Wound tissues were excised on days 3, 6, and 12, fixed in 10% neutral buffered formalin, and stained with hematoxylin-eosin or Masson’s trichrome as done by other researchers (Zandifar *et al*. 2012; Gautam and Goel 2013; Nasiry *et al*. 2022). Neutrophils, macrophages, fibroblasts, and capillary density were quantified microscopically as done by other researchers (Chen *et al*. 2017; Nasiry *et al*. 2022; Haestidyatami *et al*. 2019; Stryker *et al*. 2019). Re-epithelialization was scored using a standardized scale. Collagen density was analyzed using ImageJ software. Hydroxyproline content was determined by HPLC following acid hydrolysis as done by other researchers (Karimi *et al*. 2013 and Roodbari *et al*. 2004).

### Phytochemical and Antioxidant Analyses

Total phenolic content was measured using the Folin–Ciocalteu method, while total flavonoid content was determined by the aluminum chloride colorimetric method. Antioxidant activity was assessed using the DPPH assay, and IC_50_ values were calculated.

### LC-MS/MS and GC-MS Analyses

Chemical profiling of UCSME was performed using UPLC-QToF-MS (ESI positive and negative modes) and GC-MS. Compounds were identified by comparison with spectral libraries and expressed as relative abundances.

### Statistical Analysis

Data were analyzed using one-way ANOVA followed by Tukey HSD post-hoc test (p < 0.05). Data homogeneity was confirmed using Levene’s test.

## Results

### Plant Identification

Botanical identification conducted at the Bogoriense Herbarium (BRIN, Cibinong, Indonesia) confirmed that the plant material used in this study was *Uncaria callophylla* Blume ex Korth. (family Rubiaceae).

### Characteristics and Yield of UCSME

The *Uncaria callophylla* stem methanol extract (UCSME) appeared as a thick, sticky, blackish-green gel. Maceration of 100 g dried stem powder yielded 14.61 g of extract, corresponding to a yield of 14.61%. This value exceeds the minimum standard for herbal extracts (>12%) set by the Indonesian Herbal Pharmacopoeia, indicating efficient extraction of bioactive compounds.

### Macroscopic Evaluation of Wound Healing

**Figure 3:**
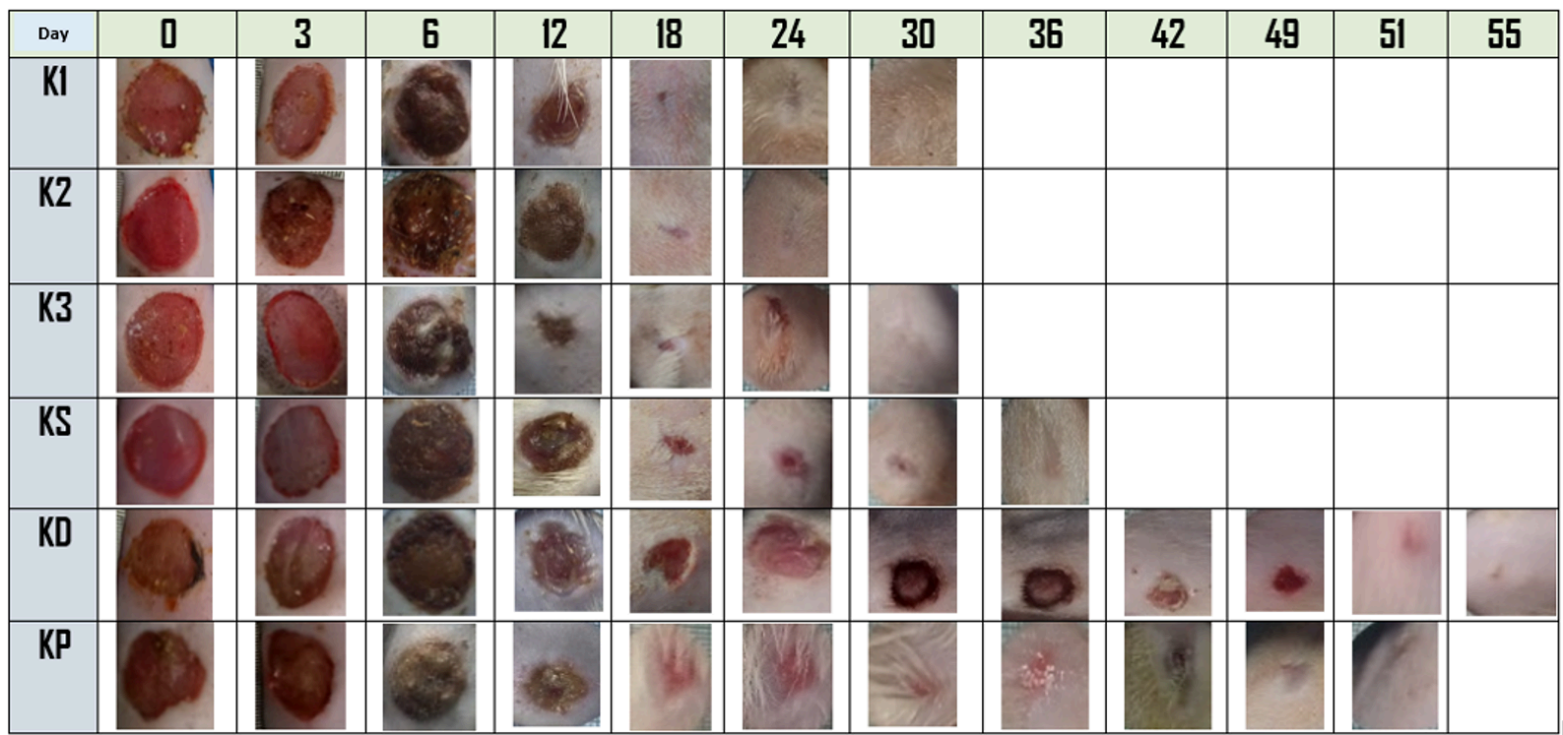
Progress of macroscopic wound healing Description: KS = Group of healthy rats that were not induced by diabetes were given ointment base KD = Group of diabetic rats given ointment base KP = Group of diabetic rats given betadine ointment K1 = Group of diabetic rats given 5% concentration of UCSME ointment K2 = Group of diabetic rats given 10% concentration of UCSME ointment K3 = Group of diabetic rats given 20% concentration of UCSME ointment

Macroscopic observation of wound healing progression is shown in Figure 3. Diabetic rats treated with UCSME ointment at concentrations of 5% (K1), 10% (K2), and 20% (K3) exhibited faster wound healing compared with untreated diabetic rats (KD) and diabetic rats treated with betadine ointment (KP). Notably, wound contraction and drying were evident as early as day 6 in all UCSME-treated groups.

By day 12, wounds in the 20% UCSME group (K3) were markedly smaller than those in untreated diabetic rats, betadine-treated diabetic rats, and even healthy control rats (KS). In contrast, wounds in the betadine-treated group remained relatively larger throughout the observation period. Overall, topical application of UCSME ointment at all tested concentrations accelerated macroscopic wound closure in diabetic rats more effectively than betadine and ointment base controls.

### Wound Area Reduction and Complete Epithelialization Time

**Figure 4:**
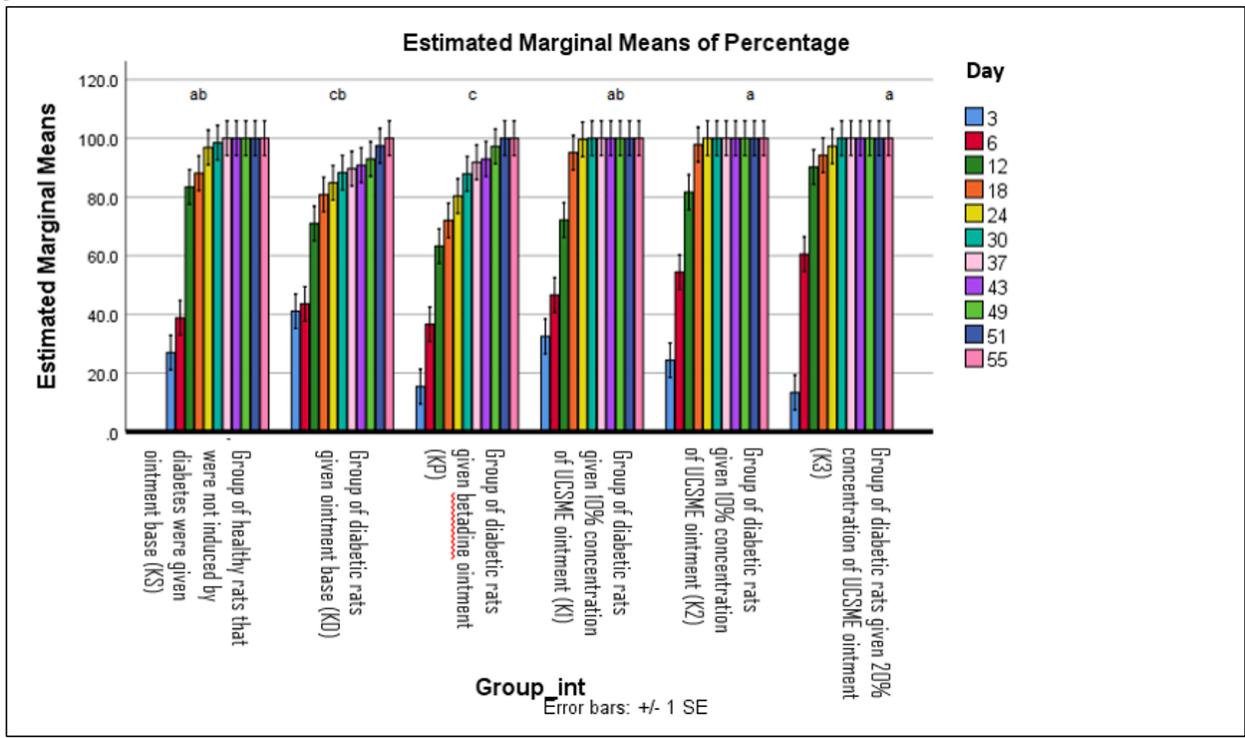
Wound Reduction

Two-way ANOVA analysis showed that treatment groups and observation time significantly affected wound area reduction (p < 0.05), with homogeneous data distribution (Levene’s test, p > 0.05). From day 3 to day 24, topical application of UCSME ointment at concentrations of 5%, 10%, and 20% significantly accelerated wound contraction compared to untreated diabetic rats (KD). However, no significant differences were observed among the three UCSME concentrations, indicating comparable effectiveness in reducing wound area (Figure 4)

**Figure 5:**
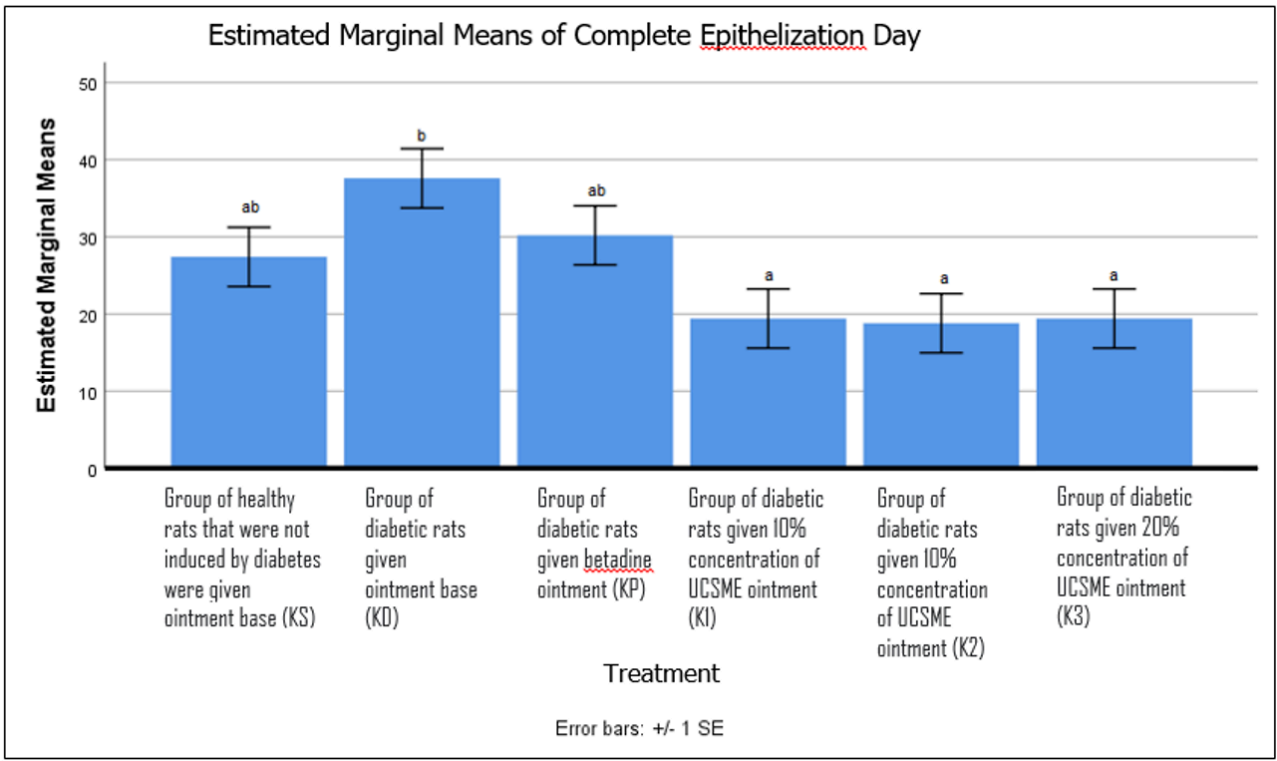
Complete Epithelialization Time

Similarly, UCSME ointment at all tested concentrations significantly shortened the time required for complete epithelialization compared to KD (p < 0.05). No significant differences were observed between UCSME-treated groups and the betadine-treated group (KP), demonstrating comparable efficacy in accelerating epithelial closure (Figure 5).

### Inflammatory Cell Response

**Figure 6:**
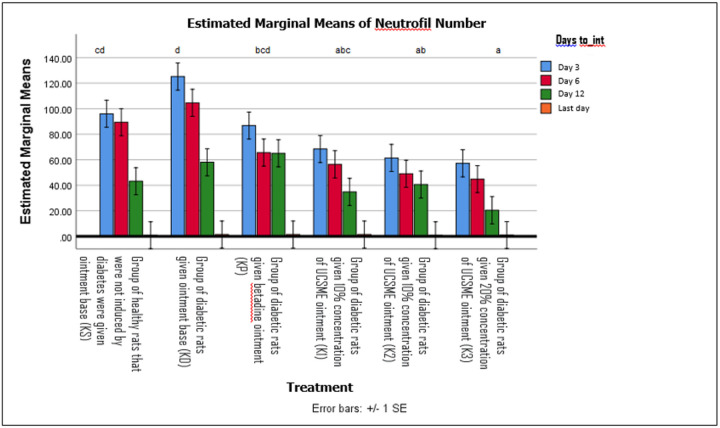
Neutrophil Number

Topical application of UCSME ointment increased neutrophil counts during the inflammatory phase. Concentrations of 10% and 20% significantly increased neutrophil numbers compared to KD and showed no significant difference from healthy controls (KS) and KP. The 5% UCSME ointment also significantly increased neutrophil counts compared to KD (Figure 6).

**Figure 7:**
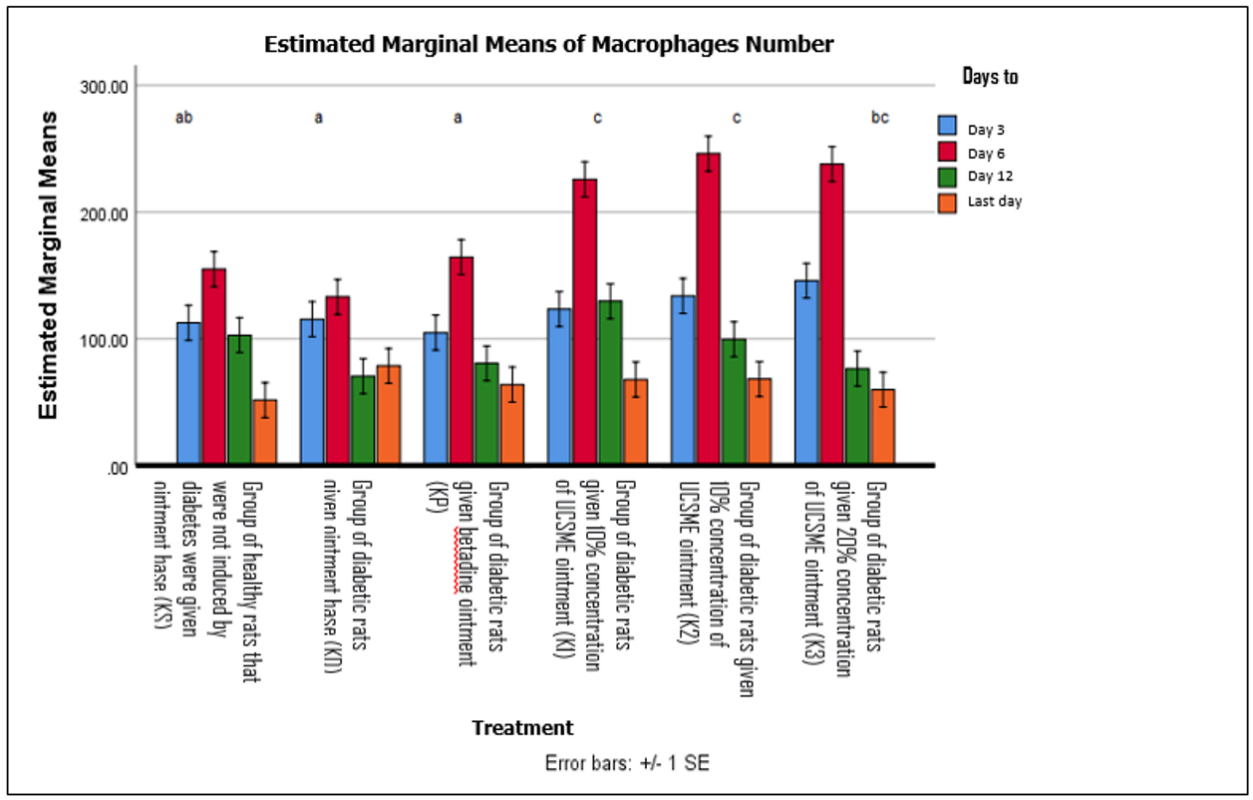
Macrophage Number

Macrophage numbers were significantly higher in all UCSME-treated groups compared to KD, KP, and KS (p < 0.05). Macrophage counts peaked on day 6 in all groups and declined thereafter, corresponding to wound progression. The highest macrophage numbers were observed in the 10% and 20% UCSME groups during the proliferative phase (Figure 7)

### Fibroblast Proliferation and Angiogenesis

**Figure 8:**
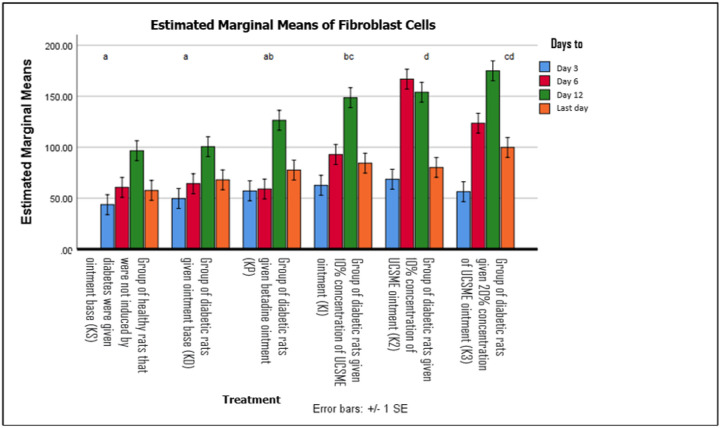
Fibroblast Number

UCSME ointment at all concentrations significantly increased fibroblast numbers compared to KD and KS. Fibroblast counts peaked on day 12, with the highest values observed in the 20% UCSME group, followed by the 10% and 5% groups (Figure 8).

**Figure 9:**
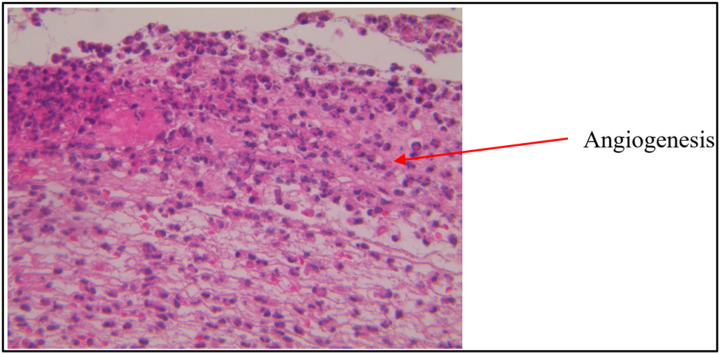
Microscopic image of wound tissue for angiogenesis calculation

**Figure 10:**
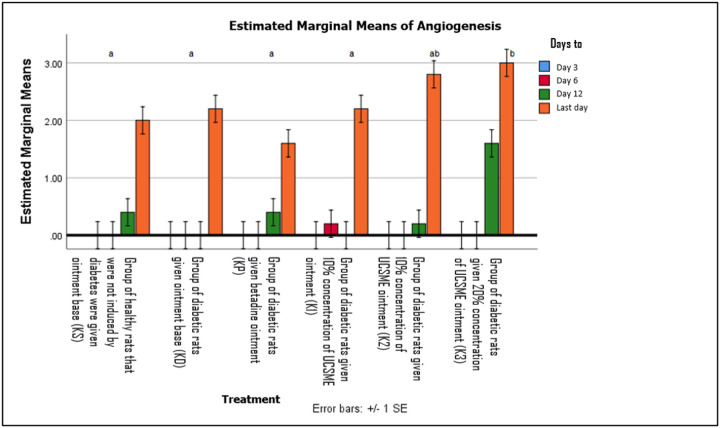
Angiogenesis Number

Angiogenesis was first observed after day 6. On day 12, angiogenesis was present in all groups except KD, with the highest angiogenesis scores observed in diabetic rats treated with 20% UCSME (Figure 9 and 10).

### Re-epithelialization, Collagen Deposition, and Hydroxyproline Content

**Figure 11:**
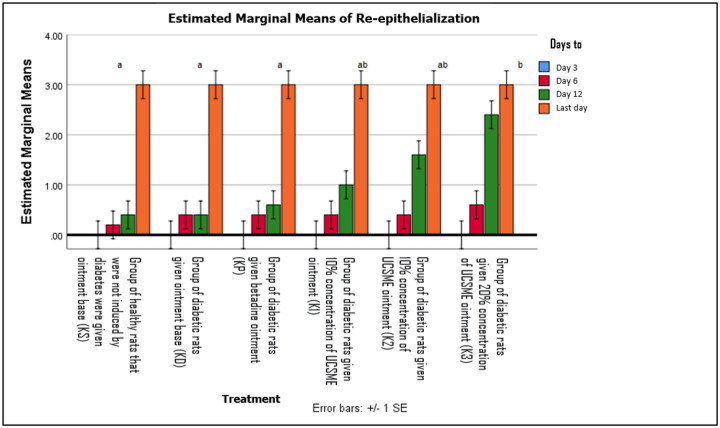
Epithelialization

Re-epithelialization began on day 6 in all groups. UCSME ointment at 20% significantly enhanced re- epithelialization compared to KD, KP, and KS, while 5% and 10% concentrations showed similar effects (Figure 11).

**Figure 12:**
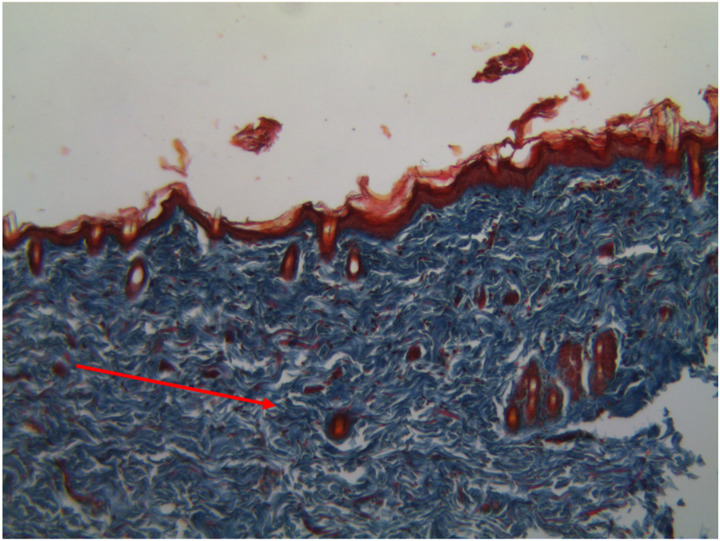
Microscopic Image of Newly Formed Collagen

Collagen fiber density was significantly increased by UCSME ointment at 10% and 20% compared to KD and KS, with the 20% concentration showing the strongest effect. Collagen density peaked on day 3 and day 12 in the 20% group and on day 6 in the 10% group (Figure 12 dan 13).

**Figure 14:**
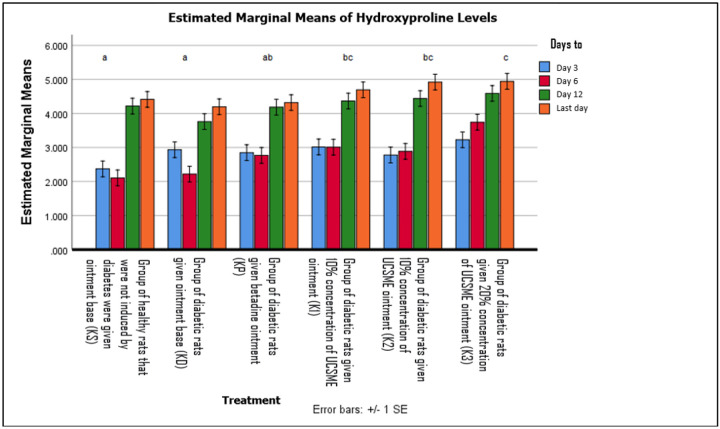
Hydroxyproline Levels

Hydroxyproline levels were significantly higher in the 20% UCSME group compared to KP, KD, and KS. However, no significant differences were observed among UCSME concentrations, indicating similar effectiveness in enhancing collagen synthesis (Figure 14).

#### Total Phenolic, Flavonoid Content, and Antioxidant Activity of UCSME

Spectrophotometric analysis using the Folin-Ciocalteu method showed that UCSME contained a high level of total phenolic compounds, amounting **to 20.59% (w/w) gallic acid equivalents (GAE)**. Quantification was based on a gallic acid calibration curve with a strong linear relationship (R^2^ = 0.9986), indicating good analytical accuracy.

Total flavonoid content of UCSME, determined using the aluminum chloride colorimetric method with quercetin as the standard, was **0.40% (w/w)**. The quercetin calibration curve exhibited good linearity, confirming reliable flavonoid quantification.

Antioxidant activity of UCSME, assessed by the DPPH free radical scavenging assay, demonstrated **very strong antioxidant capacity**, with an **IC**_**50**_ **value of 16.06 ppm**, which falls within the category of highly potent antioxidants (IC_50_ < 50 ppm). Ascorbic acid, used as a reference antioxidant, showed an IC_50_ of 4.38 ppm.

These findings indicate that UCSME is rich in phenolic and flavonoid compounds and possesses strong antioxidant activity, which may contribute to its observed wound-healing effects through modulation of oxidative stress.

### LC-MS/MS AND GC-MS Analysis of UCSME

LC-MS/MS analysis identified **five major secondary metabolites** in UCSME, namely **Arecatannin A1, Betulonic acid, Epianhydrobelachinal, Sweroside-2, and Uncarine A**. These compounds are known to possess biological activities related to **anti-inflammatory, antioxidant, vasodilatory, cytoprotective, and tissue-regenerative functions,** supporting their potential role in diabetic wound healing.

GC-MS analysis revealed the presence of **24 phytochemical constituents,** of which **nine compounds showed high similarity indices (≥90%)**. The dominant compounds included **n-hexadecanoic acid(16.63%), 1,3,7-trimethyl-3,7-dihydro-1H-purine-2,6-dione (13.07%), hexadecanoic acid methyl ester,** and several unsaturated fatty acid derivatives. These compounds are associated with **anti-inflammatory, enzyme inhibitory, antioxidant, and tissue-protective activities**.

## Discussion

Diabetic wounds represent one of the most severe complications of diabetes mellitus, characterized by delayed healing due to hyperglycemia-induced vascular dysfunction, immune dysregulation, oxidative stress, and impaired tissue regeneration (Burgess *et al*. 2021 and Spampinato *et al*. 2020). These multifactorial disturbances limit the effectiveness of conventional wound therapies and justify the exploration of plant-based alternatives rooted in ethnomedicine. *Uncaria callophylla* Blume ex Korth., traditionally used by the Dayak Ngaju community of Central Kalimantan for chronic wound treatment, represents such a promising candidate.

In the present study, topical application of *Uncaria callophylla* stem methanol extract (UCSME) ointment at concentrations of 5%, 10%, and 20% significantly improved wound healing in diabetic rats compared with untreated and betadine-treated controls. Even the lowest concentration (5%) effectively reduced wound area and shortened epithelialization time, indicating that UCSME exhibits strong biological activity at relatively low doses. Histological analyses further confirmed enhanced fibroblast proliferation, collagen deposition, and epithelial regeneration in UCSME-treated wounds.

The acceleration of wound contraction observed from day 6 onward corresponds to the proliferative phase of healing, during which fibroblast migration, extracellular matrix synthesis, and angiogenesis dominate (Velnar *et al*. 2009 and Yates, 2017). UCSME treatment promoted fibroblast activity and collagen synthesis, with peak fibroblast numbers and collagen density occurring between days 6 and 12, reflecting efficient progression from inflammation to proliferation.

UCSME also modulated the inflammatory response by increasing neutrophil and macrophage infiltration during early healing stages. Neutrophils facilitate debris clearance, while macrophages coordinate tissue repair through cytokine and growth factor release, thereby stimulating angiogenesis and fibroblast function (Yang *et al*. 2025). Angiogenesis was most pronounced in the 20% UCSME group, although all concentrations enhanced vascularization compared to controls, ensuring improved oxygen and nutrient delivery to wound tissue.

Re-epithelialization was significantly accelerated in UCSME-treated groups, with the strongest effect observed at 20%, while 5% and 10% concentrations showed comparable efficacy. This suggests a largely dose-independent effect, potentially enhanced by the moist wound environment created by the ointment base, which is known to support epithelial regeneration (Karimi *et al*. 2013).

Phytochemical profiling revealed that UCSME contains diverse bioactive compounds, including betulonic acid, uncarine A, sweroside-2, arecatannin A1, and several fatty acid derivatives. These compounds possess antioxidant, anti-inflammatory, antimicrobial, and antidiabetic properties, which collectively contribute to wound repair. High phenolic (20.59% GAE) and flavonoid (0.40% QE) contents, along with strong antioxidant activity (IC_50_ = 16.06 ppm), support the role of UCSME in mitigating oxidative stress, a key factor impairing diabetic wound healing.

Enhanced collagen fiber density and hydroxyproline levels in UCSME-treated wounds further indicate improved collagen maturation and tensile strength, essential for stable wound closure. Among the tested formulations, the 10% UCSME ointment provided the most balanced therapeutic effect, achieving robust healing responses without the potential risks associated with higher concentrations. In contrast, betadine ointment showed inferior outcomes, likely due to its cytotoxic effects on fibroblasts and collagen synthesis.

Overall, UCSME promotes diabetic wound healing by modulating inflammation, enhancing angiogenesis, stimulating fibroblast activity, accelerating epithelialization, and improving collagen deposition. These findings validate the traditional use of *Uncaria callophylla* and highlight its potential as a natural, multi-target therapeutic agent for diabetic wound management.

Topical application of *Uncaria callophylla* stem methanol extract (UCSME) ointment effectively accelerates wound healing in diabetic rats. UCSME treatment significantly enhances wound contraction, shortens complete epithelialization time, promotes angiogenesis, accelerates re-epithelialization, stimulates collagen synthesis, increases neutrophil infiltration during the inflammatory phase, and elevates macrophage and fibroblast numbers during the proliferative phase. These findings demonstrate that UCSME positively modulates multiple stages of the wound healing process.

Phytochemical analysis revealed that UCSME contains high levels of bioactive compounds, including total phenolics (20.99% w/w) and flavonoids (0.40% w/w), which are closely associated with its strong antioxidant activity (IC_50_ = 16.06 ppm). LC-MS/MS analysis identified five major secondary metabolites—betulonic acid, epianhydrobelachinal, uncarine A, sweroside-2, and arecatannin A1, while GC-MS analysis revealed nine dominant compounds with high spectral quality, including fatty acids, alkaloids, and phenolic derivatives known to possess antioxidant, anti-inflammatory, antimicrobial, and tissue-regenerative properties.

Overall, the wound healing efficacy of UCSME is attributed to the synergistic action of its diverse secondary metabolites, particularly phenolic and flavonoid compounds with strong antioxidant capacity. These results scientifically validate the traditional use of *Uncaria callophylla* in wound management and highlight UCSME as a promising natural therapeutic candidate for the treatment of diabetic wounds.

## Supplementary Material

Supplementary Files

This is a list of supplementary files associated with this preprint. Click to download.

- SupplementaryFiles.jpeg

## Figures and Tables

**Figure 1:**
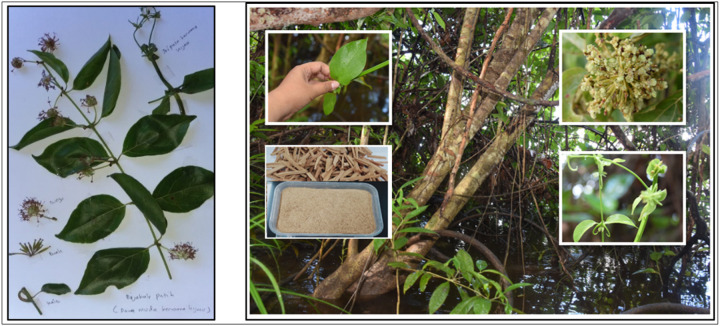
*Uncaria callophylla* Blume ex Korth.

**Figure 2:**
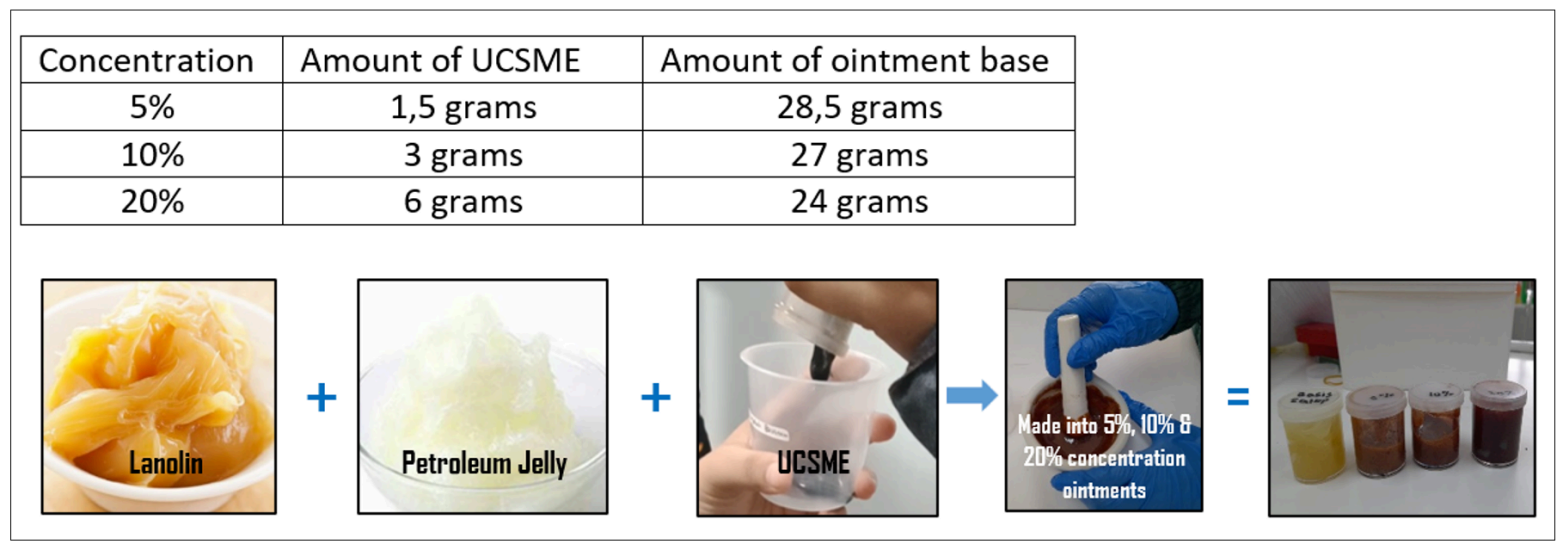
UCSME Ointmen Formulation and Preparation

**Figure 13:**
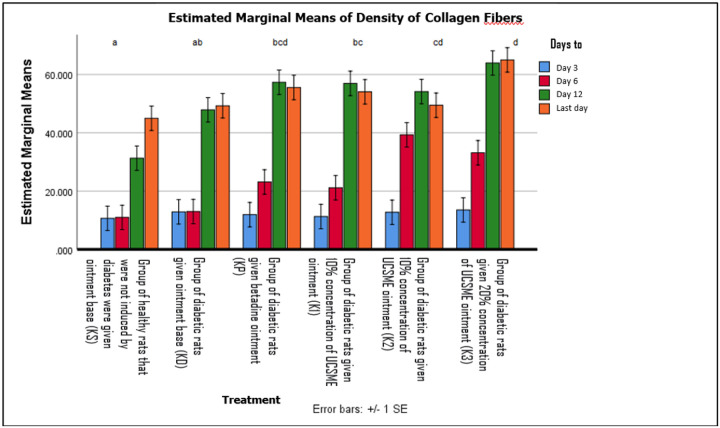
Density of Collagen Fibers

**Figure 15:**
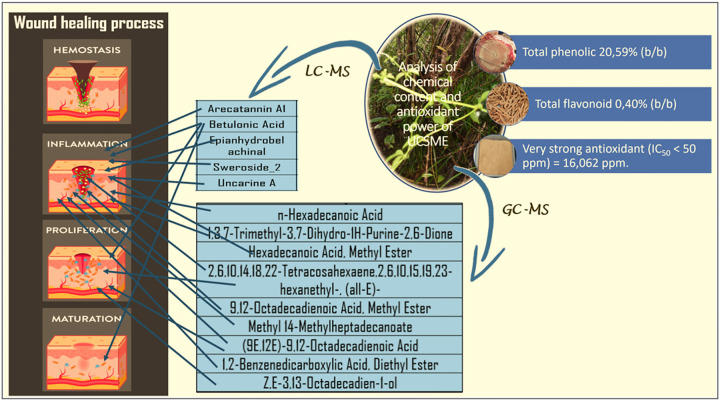
Biological potential that supports the healing process of diabetic wounds
